# Community social responsibility of continued and appropriate use of silver amalgam as dental restorative material in southern India: A cross-sectional study

**DOI:** 10.12688/f1000research.122690.4

**Published:** 2024-04-26

**Authors:** Ramprasad Vasthare, Nidambur Vasudev Ballal, Prajna P Nayak, Pujan Kamath, Nishu Singla, Thrisha Hegde

**Affiliations:** 1 Department of Public Health Dentistry, Manipal College of Dental Sciences, Manipal, Manipal Academy of Higher Education, Manipal, Karnataka, 576104, India; 2 Department of Conservative Dentistry and Endodontics, Manipal College of Dental Sciences, Manipal Academy of Higher Education, Manipal, 576104, India; 3 Indian Dental Association, Manipal, Karnataka, 576104, India

**Keywords:** dental amalgam, mercury toxicity, social responsibility, dental material, waste management, dental education, affordability

## Abstract

**Background:**

For more than 150 years, dental amalgam (DA) has been popular as a dental restorative material. Many organizations oppose its use due to perceived toxicity and environmental concerns. Hence, this study aimed to explore the continued use of DA from a South Indian dental practitioners’ perspective.

**Methods:**

This cross-sectional study was conducted among fifty-two private and public dental practitioners of Udupi district in Southern India. A self‑administered questionnaire was distributed, that involved assessment of their preferences, continuation of use and concerns of using DA as a restorative material. The percentage contribution of each variable was calculated. Preferences for continuation of use of silver amalgam based upon the age, experience and mercury toxicity as a risk factor were analyzed using Students-t test and Chi-square test.

**Results:**

Most dentists were satisfied (87%) with the results of the DA, found minimal failures (96%) and found DA more economical (89%). More than half (54%) of the participants reported that they would not continue the use of DA owing to mercury toxicity and environmental concerns. Dentists with higher age and longer clinical experience preferred continuation of DA.

**Conclusions:**

Despite satisfaction with DA for its minimal failure, longevity and affordability, the authors found that most practitioners did not prefer its continued usage. This highlights their concerns over mercury toxicity and soft tissue lesions and accentuates their community social responsibility. There is also an urgent need to educate dentists on mercury hygiene, mercury waste management and disposal.

## Introduction

Dental amalgam (DA) has been popular as a restorative material for more than 150 years particularly in large cavities, owing to tremendous mechanical properties and durability. It makes up for seventy-five percent of all dental restorations performed across the world [ Bharti *et al.*, 2010]. DA is a combination of alloy particles and elemental mercury. The usage of the “silver paste” was first found in the Chinese medical texts written by Su Kung in 659 AD [ Hsi-T’ao, 1958]. In early 1800s, D’Arcet Mineral Cement was developed in France, which is regarded as the first dental amalgam [ Magkert, 1991]. The use of room temperature mixed amalgam as a dental restorative material was formerly advocated by Bell in England (1819) and Traveau in France (1826) [ Frykholm, 1957 and Greener, 1979].

Functionally and financially, DA has been a source of great comfort for the common man. The plasticity and strength of the restorative material is a quality that has made dental practitioners utilize it not just for regular restorative work, but also for the making of dental Inlays and Onlays [ Bharti *et al.* 2010].

In recent times, we have noticed a ‘phase-down’ of DA in many parts of the world [ Espelid *et al.*, 2006; Al-Asmar *et al.*, 2019; Spencer, 2000; Brennan and Spencer, 2003] have reported reducing use of DA in recent times [ Brennan and Spencer, 2003]. In a study conducted by Al-Asmar *et al.*, shift to aesthetic restorations were seen among the dentists. In another study, DA usage was reduced, yet constituted more than half of the restorations during the 5-year period under review [ Umesi, Oremosu and Makanjuola, 2020].

Since the beginning there have been debates around its usage. In 1833, the Crawcour brothers introduced a newer version of DA “The Royal Mineral Succedaneum” to America that resulted in multiple failed amalgam restorations that sparked the “First Amalgam War” in 1845 [ Molin, 1992]. The American Society of Dental Surgeons condemned amalgam usage as malpractice and if used, member would be expelled from the society [ Mosteller, 1961]. The criticism of amalgam gradually muted with improved handling and performance of the amalgam versions put forth by Elisha Townsend, J Foster Flagg and G.V. Black [ Flagg, 1843; Cannon *et al.*, 1985]. The “Second Amalgam War” resulted from the writings of Dr Alfred Stock, who was poisoned with mercury through the twenty- five years of exposure to the metal [ Weiner, Nylander and Berglund, 1990]. A committee was appointed to study allegations, which concluded that amalgam has a rightful place in dentistry and that there was no reason to stop its use [ Eames, 1959]. The current controversy popularly known as “Third Amalgam War” stemmed from the words of HA Huggins in 1973 who suspected that everything from leukemia to bowel disorders could be due to patient’s reaction to mercury [ Huggins, 2007]. The Consumer Report of 1986 exposed this anti - amalgam movement that subsided this controversy. Again, the “60 Minutes” TV program re-intensified the issue again thereby creating considerable public alarm [ Dodes, 2001].

Mercury is present in abundance in the natural environment and a substantial number of people are exposed to it in various ways [ Dodes, 2001]. Yet, any symptoms of unknown etiology have been frequently linked to water fluoridation and dental amalgam restorations [ Boyd *et al.*, 1991; Huggins, 2007].

In recent times, various organizations around the world have attempted to reduce the usage of DA. Its usage is brought down after the Minamata convention, a global health and environment treaty that governs the mining, usage and trade in mercury. It entreats for “phase down of dental fillings using mercury amalgam”. It included various strategies like “aiming at dental caries prevention and use of mercury-free dental restoration alternatives and on promoting best management practices. As well as promoting the use of best environmental practices in dental facilities to reduce release of mercury and mercury compounds to water and land” [ Minamata Convention, 2014].

The European Commission’s Scientific Committee on Health and Environmental Risks (“SCHER”) authenticates that “dental amalgam in the environment can methylate (forming methylmercury, which is the most toxic form of mercury)” [ SCHER, 2014]. In 2015 the European Commission’s Scientific Committee on Emerging and Newly Identified Health Risks (“SCENIHR”) changed their stance from amalgam is “a safe and effective restorative material” to that amalgam is only “an effective restorative material” [ SCENIHR, 2014, 2015]. Based on these, the European Union has accorded the “Berlin declaration” in 2017 to end amalgam use in Europe by 1 July 2022 [ Berlin Declaration, 2017].

In contrast, there is vast research done that supports DA usage and provides scientific evidence for its safety [ Heggland *et al.*, 2011; Melchart *et al.*, 2008; Woods *et al.*, 2008]. Many other organizations too contradict the above standpoint of SCHER and SCENIHR [ U.S. Food and Drug Administration, 2020; National Institute of Health, 2006; Alzheimer’s myths; Uçar and Brantley, 2017]. No correlations were found between exposure to DA and neuropsychological and renal functions in many randomized trials done all over the world [ Bellinger *et al.*, 2006; Barregard, Trachtenberg, and McKinlay, 2008; Lauterbach *et al.*, 2008]. A systematic review and meta-analysis conducted by Aminzadeh and Etminan did not provide evidence for or against an association between the presence of DA restorations and multiple sclerosis [ Aminzadeh and Etminan, 2007].

Studies did not find any association between urinary mercury concentrations among dentists and dental nurses with self-reported memory disturbance or mercury vapors in the dental office with cytogenetic damage to leukocytes [ Ritchie *et al.*, 2004; Atesagaoglu *et al.*, 2006].

The U.S. Food and Drug Administration states, “We have reviewed the best available scientific evidence to determine whether the low levels of mercury vapors associated with dental amalgam fillings are a cause for concern. Based on this evidence, U.S. Food and Drug Administration considers dental amalgam fillings safe for adults and children aged six and above. Clinical studies in adults and children ages six and above have found no link between dental amalgam fillings and health problems” [ U.S. Food and Drug Administration, 2020]. The National Institute of Dental and Craniofacial Research in the U.S. Dept. of Health and Human Services also states that children whose cavities were filled with dental amalgam had no adverse health effects. The findings included no detectable loss of intelligence, memory, coordination, concentration, nerve conduction or kidney function during the 5-7 years the children were followed” [ National Institute of Health, 2006].

Given the dual stance of dental amalgam usage by various organizations around the world, and notable reduction in DA usage over the years by dental practitioners, we wanted to explore the continued usage of silver amalgam as a tooth restorative material, from South Indian dental practitioners’ perspectives in an unbiased manner.

## Methods

### Ethics statement

This study was initiated after approval from the Kasturba Medical College and Kasturba Hospital Institutional Ethics Committee [dated 12/2018; Reference No. 569], which is the ethical committee for MAHE University. The study was conducted in agreement with the World Medical Association Declaration of Helsinki, 1975. Prior to the start of study, a signed informed consent for participating in the study and reuse of anonymized data was received from each participant.

### Study design

A cross-sectional study was conducted over a duration of sixteen weeks in 2019 from second week of January to second week of May across various dental practices in the district of Udupi in alliance with Indian Dental Association, Udupi district branch, in Southern India. The inclusion criteria for the study participants were (i) Government or private practitioners (ii) Dental practitioners who have the willingness and who consented to participate in the study. The exclusion criteria for the study were: (i) those practicing for less than 5 years; and (ii) inability/unwillingness to participate in the study.

A total of one hundred and thirty-four (134) dental practices encompassing all the seven ‘Taluks’ were identified. Since it was a small population size, a complete enumeration of the study population was done. They were assessed on questions relating to their opinions of using DA.

Private dental clinics: The list of registered practitioners in Udupi district as per the Karnataka State Dental Council Registration list was used as a reference document for contacting the clinics individually. Access to this list was granted after the authors submitted a request to the Karnataka State Dental Council. Names and mail addresses were also obtained from the list of the largest non-governmental dental organization in India, namely the Indian Dental Association, Udupi branch. Access to this list was granted after the authors submitted a request to the Indian Dental Association.

Public health care centers: Oral health delivery in public sector is unified into the existing public hospital setups and is available from community health centers and district hospitals. So, we included practitioners from six Community Health Centers (CHCs) and the district hospital, details of the same obtained from official portal of Karnataka State Health Ministry (District wise Hospital details with facilities).

After excluding those not fulfilling the inclusion criteria, ninety-two (92) participants were available for the study. Due to the small population size, we included all the practitioners, public and private, in the study.

Questionnaires were distributed through email and communication network of IDA Udupi District branch. We attempted to contact non-respondents during the conduct of four Continuing Dental Education programs for dental practitioners to ensure maximum participation of respondents.

A self-administered questionnaire [ Nayak, 2022] assessing the practitioners’ preferences, continuation of use and concerns of using DA as a restorative material was developed based on similar studies [ Brennan and Spencer, 2003; Maciel *et al.*, 2017; Espelid *et al.*, 2006]. Four subject experts checked the face validity and content validity of questions and finalized the questionnaire. A pilot survey was conducted among 15 dentists working in an academic setting to confirm the needed background preparations and clarity of specific terms in questionnaire that could seem unclear. The findings and responses of pilot study were found to be favorable, facilitating the initiation of the larger planned study. Their responses, however are not included in the study results.

The questionnaire consisted of two sections: (a) Four questions on respondents’ demographic and professional particulars: age, gender, qualification and type of practice and (b) fourteen closed-ended questions regarding duration of DA usage and preferences for DA over other restorative materials, preferred type of cavity for DA usage, experiences regarding ease of use, longevity, failures, soft tissue lesions and mercury toxicity during DA usage as well as on patient affordability and satisfaction of DA.

### Statistical analysis

Responses were documented on Microsoft Excel and data analysis was performed using IBM Statistical Package for the Social Sciences (SPSS) version 26 (IBM Corp., Armonk, N.Y., USA). The percentage contribution was obtained for each significant variable. Preferences for continuation of use of silver amalgam based upon the age of dentists has continuous variables, so analysis was done using Students t-test. Since the sample was small, Shapiro Wilk test was done was performed, which did not show evidence of non-normality (W = 0.78, p-value = 0.26). Results of preferences for the use of DA by practitioners, duration and satisfaction of usage of DA, experiences regarding longevity, their perceptions on the risks associated with DA and patient satisfaction of restorations were analyzed using chi-square test at 5% level of significance.

## Results

As the population size was small, a complete enumeration of all the 134 dental practitioners was done. Of these, only Ninety-two practitioners fulfilled the inclusion criteria, and hence were included in the study. Out of them, 52 dental practitioners responded (Response rate 55.9%). About the gender distribution, there were equal number of male and female participants (26 out of 52 each). The mean age group of study population was 34.9 years.

Table 1 describes the experience and satisfaction for DA use among practitioners. About 77% of participants reported that they have been using DA for less than ten years and 87% participants were very satisfied/satisfied with its use. The longevity of DA is highly appreciated by the dental practitioners as 44% and 48% participants found the longevity “Very Good” and “Good” respectively. Most of them reported that their patients were ‘very satisfied’ and ‘satisfied’ (76.9% and 15.4% respectively) of the DA restorations.

**Table 1.** Experience and satisfaction for DA (dental amalgam) use among practitioners.

	Variable		N (%)
1	Duration of usage of silver amalgam	Less than 10 years	40 (76.9%)
		10 to 20 years	9 (17.3%)
		20 to 30 years	2 (3.8%)
		30 or more years	1 (1.9%)
2	Satisfaction for usage of silver amalgam	Very satisfied	15 (28.8%)
		Satisfied	30 (57.7%)
		Dissatisfied	7 (13.5%)
		Very dissatisfied	0
3	Experience regarding longevity of the restoration	Very good	23 (44.2%)
		Good	25 (48.1%)
		Fair	4 (7.7%)
		Poor	0
4	Experience regarding patient satisfaction for silver amalgam	Very satisfied	8 (15.4%)
		Satisfied	40 (76.9%)
		Dissatisfied	4 (7.7%
		Very dissatisfied	0
5	Experience regarding failure of restoration	Very minimal (small) number	15 (28.8%)
		Minimal (small) number	35 (67.3%)
		Large	1 (1.9%)
		Very large	1 (1.9%)
6	Experience regarding any soft tissue lesions due to silver amalgam	Yes	21 (40.4%)
		No	31 (59.6%)
		**Total**	**52 (100%)**

Table 2 describes the opinions and preferences of practitioners for DA usage. Regarding the ease of use, equal number (48% each) of participants found it “easy to use” and “difficult and cumbersome”. When asked about the type of cavity for which they would prefer to use DA, 46 (59.6%) participants responded that they would use it for medium and large cavities. They also reported that DA is the material of choice economically as 89% of participants found it economical and 98% found it affordable by patients. Yet, 54% of the participants reported that they would not suggest the use of DA compared to other tooth-colored restorations.

**Table 2.** Opinion and preference for the use of DA (dental amalgam) by practitioners.

	Variable		N (%)
1	Opinion regarding ease of use of silver amalgam	Very easy	2 (3.8%)
		Easy and comfortable	25 (48.1%)
		Difficult and cumbersome	25 (48.1%)
		Very difficult	0
2	Preference for silver amalgam use based on size of the cavity	Very large	5 (9.6%)
		Large	26 (50%)
		Medium	21 (40.4%)
		Small	0
		Very small	0
3	Material of choice - economically	Very economical	6 (11.5%)
		Economical	40 (76.9%)
		Not economical	6 (11.5%)
4	Affordability for patients	Very affordable	9 (17.3%)
		Affordable	42 (80.8%)
		Unaffordable	1 (1.9%)
5	Opinion regarding continuation of use/suggest usage than other tooth-colored materials	Yes	13 (25%)
		No	28 (53.8%)
		Not sure	11 (21.2%)
		**Total**	**52 (100%)**

Table 3 elucidates the perception and awareness for DA use as a risk factor. It was found that nearly 94% were aware of mercury toxicity concerns. Moreover, 46% and 37% participants felt that using DA as a restorative material could pose a risk factor for pregnant women and children respectively.

**Table 3.** Perception and awareness for DA (dental amalgam) use as a risk factor among practitioners.

	Variable		N (%)
1	Perception for silver amalgam use as a risk factor for pregnant women	Yes	24 (46.2%)
		No	11 (21.2%)
		Not sure	17 (32.7%)
2	Perception of silver amalgam use as a risk factor for Children	Yes	19 (36.5%)
		No	15 (28.8%)
		Not sure	18 (34.6%)
3	Concerns about mercury toxicity on environment	Yes	49 (94.2%)
		No	2 (3.8%)
		Not sure	1 (1.9%)
		**Total**	**52 (100%)**

Preference for continued usage of DA based upon the age of dentists, showed statistically significant differences, with older practitioners preferring DA more ( Table 4). Likewise, a significantly greater number of experienced practitioners preferred continued use of DA as well as being very satisfied with DA usage ( Table 5).

**Table 4.** Preference for continuation of use of silver amalgam based upon the age of dentists.

Variable		Age (Mean ± SD)	95%confidence interval of the difference		N	P value
			Lower	Upper		
Preference	Yes	40.7 ± 10.8	3.42	12.53	14	**.001**
	No	32.7 ± 5.5	1.54	14.41	38	
				**Total**	**52**	

p value derived by student t test, p < 0.05 considered significant.

**Table 5.** Preference for continuation of use of silver amalgam based upon experience of dentists.

Variable		Preference % (N)		P value
		Yes	No	
Duration of usage of silver amalgam	5 to 10 years	15% (6)	85% (34)	**.002**
	10 to 20 years	55.6% (5)	44.4% (4)	
	20 to 30 years	100% (2)	0	
	30 years or more	100% (1)	0	
Satisfaction with usage of silver amalgam	Very satisfied	80% (12)	20% (3)	**<0.001**
	Satisfied	3.3% (1)	96.7% (29)	
	Dissatisfied	14.3% (1)	85.7% (6)	
Experience regarding longevity of the restoration	Very good	47.8% (11)	52.2% (12)	**0.008**
	Good	8% (2)	92% (23)	
	Fair	25% (1)	75% (3)	
Experience regarding patient satisfaction for silver amalgam	Very satisfied	75% (6)	25% (2)	**0.004**
	Satisfied	17.5% (7)	82.5% (33)	
	Dissatisfied	25% (1)	75% (3)	
**Total**		**26.9% (14)**	**73.1% (38)**	

p value derived by chi square test, p < 0.05 considered significant.

## Discussion

Silver amalgam as a dental restorative material has survived time and has successfully competed with various tooth-coloured restorations in the market [ Roulet, 1997; Antony *et al.*, 2008]. The longevity of the restoration, an extensive record of minimal failures in addition to being one of the most economic dental materials in the market, makes dentists and patients opt for the product especially in developing nations like India [ Ukrainian Religious Studies, 1996; Maciel *et al.*, 2017; Peretz and Ram, 2002]. Hence, this study was conducted to explore the continued use of silver amalgam for dental restorations, from a South Indian dental practitioner’s perspective in an unbiased manner.

In our study we witnessed that although most dentists were satisfied with longevity, minimal failures of DA restorations and cost-effectiveness, more than 50% of them reported that they would not suggest dental amalgam over other tooth-colored restorations. Reported continuation of DA usage is lesser in our study as compared to previous studies [ Maciel *et al.*, 2017; Peretz and Ram, 2002]. This can be owed to their concerns over mercury toxicity, as 94% of practitioners were aware of its impact on the environment. Presence of environmental Mercury results in microbial antibiotic resistance, which in turn propounds health risk to humans and animals [ Rahman and Singh, 2018]. This is in line with Minamata Convention that calls for reduction of DA usage [ Minamata Convention, 2014]. Moreover, it was found from our study that tooth-colored restorations were preferred mostly by younger dentists as compared to older practitioners which could be due to comfortable working time and better aesthetics.

In contrast, more dentists with higher age and longer clinical experience preferred continuation of DA. This could be due to greater experience and confidence in handling DA. Other reasons for their preferences for DA usage could be cost effectiveness, minimal failures and patient affordability. These findings are comparable with other studies conducted by Maciel *et al.* [2017], Espelid *et al.* [2006] and Peretz and Ram [2002]. In our study, more than half of them preferred DA usage in medium and large cavities. This is in accordance with studies that show ineffectiveness of DA in very large cavities owing to higher chances of overhanging margins in proximal restorations [ Ghulam and Fadel, 2018]. This could be due to the exceptional mechanical properties of DA over others as well as aggravation of pain and sensitivity with composite restorations in deeper cavities. However, a shift in the concept of ‘extension for prevention’ to a modern ‘minimally invasive approach’ with newer self-adhesive materials has further reduced the use of DA. This could be the reason for significantly lower preference for DA among younger practitioners. Further, not being aesthetic as compared to other restorations, DA also causes local soft tissue lesions like amalgam tattoo and lichenoid reaction and can trigger hypersensitivity and autoimmune disorders [ Ghulam and Fadel, 2018]. This was like the results in our study, where 40% of practitioners had experienced soft tissue lesions due to DA restorations.

We observed that in the government run CHCs, none of the dentists are currently using DA. This can be attributed to (i) low rates of dental auxiliary recruitment at the CHCs, who are very much needed for handling of DA. (ii) non-availability of amalgam triturators in the government hospitals, (iii) Government’s support for the Minamata convention. There is also an attempt to move towards phase-down of DA restorations in the governmental sector.

Moreover, the study results show that the practitioners lacked extensive knowledge on mercury toxicity, as not many of them felt that they could pose a risk factor for pregnant women and children. However, more than 90% of them expressed their concerns over mercury toxicity on environment. This highlights their concerns over mercury toxicity and soft tissue lesions and accentuates their community social responsibility. That is, even though DA demonstrates cost effectiveness, minimal failures and patient affordability, dental practitioners are reducing its usage. This substantiates the dental practitioners’ awareness of community well-being and environmental safety. Yet, as older practitioners still preferred DA, there is a need to sensitize them to the precautions to be taken on mercury hygiene as well as mercury waste management, its environmental effects, and guidelines of Minamata Convention. This also necessitates adopting and disseminating evidence on the use of dental restorative materials through continuing education programs.

Consequently, as our study and various authors [ Al-Asmar *et al.*, 2019; Umesi, Oremosu and Makanjuola, 2020] report, a majority of dental practitioners across the globe do not advise the use of DA over other tooth-coloured restorative materials. There is a prevailing notion that placing dental amalgam restorations can cause adverse health effects like impairing kidney function [Eggleston, 1994 and decreasing T-lymphocyte counts [ Boyd *et al.*, 1991], although studies by Berglund [1990], University of Umea in Sweden found no evidence of kidney impairment in subjects with amalgam restorations.

Also, the hype generated from the three Amalgam Wars [ Molin, 1992; Weiner, Nylander and Berglund, 1990; Huggins, 2007] raised considerable concerns about mercury toxicity amongst the patients and dentists, in spite of being disproved repeatedly. This discussion is still a relevant debate and different countries have laid down their guidelines regarding the use or restriction of amalgam as a dental restoration with many places where amalgam phase down is moving from a debatable domain to a legislative domain [ Al-asmar *et al.*, 2019; Umesi, Oremosu and Makanjuola, 2020].

However, our study had certain limitations; a significant reduction in sample size occurred due to a minimum of five years of experience as inclusion criteria. Also, certain practitioners had completely shifted to tooth-coloured restorations, precluding them from the study. Among those included in the study, a significant number of participants did not respond, despite repeated reminders, citing busy patient schedules, making it a limitation of this study.

## Conclusions

To draw inferences from this study, it is important to take a calculated decision while selecting the right restorative material based on individual case scenario and economics of the patient. India is a developing country where emphasis on oral health is minimal, and majority of the population is incapable of meeting increased expenses on dental treatment. Hence, dental amalgams still stand as a good restorative material for low-middle income countries.

However, there is an urgent need to educate dentists about the precautions to be taken on mercury hygiene as well as mercury waste management and disposal, which by itself can help in reducing mercury toxicity and subsequent effects. There is an imperative need to sensitize the dentists on the guidelines of Minamata Convention. Patients too have to be alerted about the dental materials based on evidence so that they do not instinctively believe in biased publicity of products.

The continued and appropriate use of DA is a decision that needs careful consideration in the times of newer dental cements and the spectrum of Composites. DA is a material that has been tried and tested. The newer materials too will face the test of time and will have to prove their efficacy in the coming decades.

## Data availability

### Underlying data

Open Science Framework: Community Social Responsibility of continued and appropriate use of Silver Amalgam as dental restorative material in Southern India. https://doi.org/10.17605/OSF.IO/NUC5J [ Nayak, 2022].

This project contains the following underlying data:

- Udupi Amalgam study.xlsx (social responsibility of silver amalgam usage as a dental restorative material).

### Extended data

Open Science Framework: Community Social Responsibility of continued and appropriate use of Silver Amalgam as dental restorative material in Southern India. https://doi.org/10.17605/OSF.IO/NUC5J [ Nayak, 2022].

This project contains the following extended data:

- Questionnaire – Copy.docx

Data are available under the terms of the Creative Commons Zero “No rights reserved” data waiver (CC0 1.0 Public domain dedication).
