# Supplementary material for: Community social responsibility of continued and appropriate use of silver amalgam as dental restorative material in southern India: A cross-sectional study
Source: F1000Res. 2024 Jun 27;11:1061. Originally published 2022 Sep 16. [Version 5] doi: 10.12688/f1000research.122690.5 (PMC11167699; doi:10.12688/f1000research.122690.5)
Supplement: Peer review history

# Reviewer report: Reviewer response for version 5

**DOI:** 10.5256/f1000research.168172.r296214

**Published:** 2024-07-29

**Authors:** Nourdine Attiya

**Affiliations:** 1 Moulay Ismail University, Meknes, Morocco

Dear Authors,

 Thank you for your continued efforts in revising the manuscript titled "Community Social Responsibility of Continued and Appropriate Use of Silver Amalgam as Dental Restorative Material in Southern India: A Cross-Sectional Study." While significant improvements have been made, there are critical areas that still require attention to enhance the quality and comprehensiveness of the manuscript.

**Key Points for Further Revision:**

1. **Finite Population Correction (FPC):**
  - **Current Status:** The manuscript lacks the application of the finite population correction (FPC) despite known population and sample sizes. The response mentioned difficulty in sample size estimation, which seems unrelated to applying FPC.
  - **Recommendation:** Please clarify the rationale behind not using FPC, particularly given its importance in small populations. If applicable, incorporate FPC into the descriptive statistical analysis to improve the precision and credibility of your results.
2. **Focus on Dental Amalgam and Its Health Impacts controversies:**
  - **Current Status:** The manuscript currently emphasizes the official position on dental amalgam without a comprehensive review of its health impacts.
  - **Recommendation:** The literature review should include a thorough discussion on the health effects of mercury exposure from dental amalgam, especially concerning dental personnel. It is important to acknowledge studies that highlight potential risks, even if they are not aligned with official positions. Some relevant studies (from many others)  include:
    - Florina Andreescu C, 2017 (Ref 1)
    - Neghab M, et. al., 2011 (Ref 2)
    - Moen B, et. al., 2008 (Ref 3)
  - A balanced view of the risks and benefits associated with dental amalgam use will enhance the manuscript's comprehensiveness and relevance and guarantees its neutrality.
3. **Clarity and Repetition:**
  - **Recommendation:** Avoid repetition and ensure clarity in the language. For example, the phrase "Shapiro-Wilk test was done was performed" should be corrected to "the Shapiro-Wilk test was performed."

Is the work clearly and accurately presented and does it cite the current literature?

No

If applicable, is the statistical analysis and its interpretation appropriate?

No

Are all the source data underlying the results available to ensure full reproducibility?

Yes

Is the study design appropriate and is the work technically sound?

Yes

Are the conclusions drawn adequately supported by the results?

Yes

Are sufficient details of methods and analysis provided to allow replication by others?

No

Reviewer Expertise:

Epidemiology, Environmental health, Biostatistical modeling

I confirm that I have read this submission and believe that I have an appropriate level of expertise to confirm that it is of an acceptable scientific standard, however I have significant reservations, as outlined above.

## References

[1] : Neurotoxic effects of mercury exposure for dental workers - A literature review. *Dental, Oral and Craniofacial Research* .2017;3(4) : 10.15761/DOCR.1000209 10.15761/DOCR.1000209

[2] : Symptoms of intoxication in dentists associated with exposure to low levels of mercury. *Ind Health* .2011;49(2) : 10.2486/indhealth.ms1214 249-54 10.2486/indhealth.ms1214 21173523

[3] : Neurological symptoms among dental assistants: a cross-sectional study. *J Occup Med Toxicol* .2008;3: 10.1186/1745-6673-3-10 10 10.1186/1745-6673-3-10 18485237 PMC2427043

# Reviewer report: Reviewer response for version 5

**DOI:** 10.5256/f1000research.168172.r296213

**Published:** 2024-07-29

**Authors:** Ramkumar Yadav

**Affiliations:** 1 Department of Biomaterials Science, College of Dentistry,, Dankook University, Yongin-si, Gyeonggi-do, South Korea

All comments have been answered by the authors.

 No further comments.

Is the work clearly and accurately presented and does it cite the current literature?

Yes

If applicable, is the statistical analysis and its interpretation appropriate?

Yes

Are all the source data underlying the results available to ensure full reproducibility?

Yes

Is the study design appropriate and is the work technically sound?

Yes

Are the conclusions drawn adequately supported by the results?

Yes

Are sufficient details of methods and analysis provided to allow replication by others?

Yes

Reviewer Expertise:

NA

I confirm that I have read this submission and believe that I have an appropriate level of expertise to confirm that it is of an acceptable scientific standard.

# Reviewer report: Reviewer response for version 4

**DOI:** 10.5256/f1000research.164158.r275665

**Published:** 2024-06-10

**Authors:** Ramkumar Yadav

**Affiliations:** 1 Department of Biomaterials Science, College of Dentistry,, Dankook University, Yongin-si, Gyeonggi-do, South Korea

Why did authors work on DA materials still. 

 Add Dental composite history also.

 Add current limitations of work.

 Add futute scope of this work.

 Why did authors not perform mechanical test.

 Why did authors not perform physical test.

 Why did authors not perform thermal test.

 Why did authors not perform wear test.

Is the work clearly and accurately presented and does it cite the current literature?

Yes

If applicable, is the statistical analysis and its interpretation appropriate?

Yes

Are all the source data underlying the results available to ensure full reproducibility?

Yes

Is the study design appropriate and is the work technically sound?

Yes

Are the conclusions drawn adequately supported by the results?

Yes

Are sufficient details of methods and analysis provided to allow replication by others?

Yes

Reviewer Expertise:

Dental composite materials

I confirm that I have read this submission and believe that I have an appropriate level of expertise to confirm that it is of an acceptable scientific standard, however I have significant reservations, as outlined above.

# Response

**Published:** 2024-06-20

**Authors:** Prajna Nayak

**Affiliations:** Manipal Academy of Higher Education, India

**Reviewer 3 report and Author responses:**

**Why did authors work on DA materials still.**

**Response: **We thank the reviewer for this relevant question. A survey among dental practitioners regarding continued usage of DA is relevant in India as dental amalgam is still widely used across the Indian subcontinent. The extensive usage of DA in many parts of India prompted us in conducting this survey to understand their perception of continued usage as well as their concerns on mercury toxicity.

**Add Dental composite history also.**

**Response: **We thank the reviewer for the suggestion, but we wish to mention that the introduction is already very long and a detailed history about the dental amalgam, which is in accordance with the aim of this study, is already mentioned. Hence we will not be able to add history on dental composites.

**Add current limitations of work.**

**Response: **We have added the limitations of this study at the end of the discussion section in the page #13.

**Add future scope of this work.**

**Response: **Thank you Sir for this suggestion we have now added this in second paragraph of the conclusion section in the page #13.

**Why did authors not perform mechanical test.**

**Why did authors not perform physical test.**

**Why did authors not perform thermal test.**

**Why did authors not perform wear test.**

**Response: **Respected Sir, this was only a survey done using a questionnaire, among the dental practitioners about their perception on dental amalgam usage. Hence no physical, mechanical or wear tests were done.

# Reviewer report: Reviewer response for version 4

**DOI:** 10.5256/f1000research.164158.r269658

**Published:** 2024-05-28

**Authors:** Nourdine Attiya

**Affiliations:** 1 Moulay Ismail University, Meknes, Morocco

Dear Authors,

 I hope this message finds you well. Thank you for your diligent efforts in revising the manuscript titled "Community Social Responsibility of Continued and Appropriate Use of Silver Amalgam as Dental Restorative Material in Southern India: A Cross-Sectional Study." After thoroughly reviewing the revised version, I appreciate the improvements made so far. However, several methodological concerns need to be addressed to ensure the robustness and transparency of your study.

**Key Points for Further Revision:**

1. **Population Size and Sampling Method**:
  - **Current Status**: The revised methods section includes details on the small population size and the use of complete enumeration.
  - **Recommendation 1**: Explicitly state any limitations related to sample size, potential biases, and the generalizability of findings in a dedicated paragraph in the discussion section.
  - **Recommendation 2**: **Finite Population Correction (FPC)**: If applicable, include the finite population correction factor in the descriptive statistical analysis section. This adjustment is crucial for small populations and should be transparently reported to strengthen the credibility of the findings.
2. **Statistical Analysis Transparency**:
  - **T-Test and Chi-Square Test Assumptions**:
    - **T-Test**: The manuscript now includes information on the assumptions for the t-test.
    - **Chi-Square Test**: The assumptions for the chi-square test, particularly the need for independence of observations and the requirement that expected frequencies be at least 5, have not been discussed.
  - **Recommendation**: Please discuss how the assumptions for both the t-test and chi-square test were verified. For the chi-square test, specifically confirm that all expected frequencies met the minimum requirement.
3. **Confidence Intervals for Descriptive Statistics**:
  - **Current Status**: The response indicates a misunderstanding about the applicability of confidence intervals for proportions. My suggestion was to estimate CIs for the descriptive statistics summarized in the first three tables, not specifically for hypothesis tests.
  - **Recommendation**: Confidence intervals should be calculated and presented for the descriptive statistics (proportions and means) summarized in Tables 1-3. This will enhance the interpretability and reliability of your estimates. Confidence intervals can and should be calculated for proportions and are a standard practice in statistical analysis to express the precision of an estimate. Providing confidence intervals for proportions, and not only means, would strengthen the presentation of your results by offering a range within which the true population proportion is likely to lie. This enhances the interpretability and reliability of your findings.

If there are specific reasons or constraints that will prevent you from including confidence intervals for proportions, it would be helpful to elaborate on these in the manuscript. Otherwise, I strongly recommend incorporating confidence intervals for the proportions presented in your results section to improve the completeness and robustness of your statistical analysis.

**Conclusion:**

 While the manuscript has shown significant improvements, addressing the above points will further enhance its methodological rigor and overall quality. Including discussions on the chi-square test assumptions, providing confidence intervals for descriptive statistics, and ensuring detailed discussion on the study limitations will make your manuscript more robust and transparent.

 Thank you for your attention to these points. I look forward to seeing the revised version of your manuscript.

 Best regards,

Is the work clearly and accurately presented and does it cite the current literature?

No

If applicable, is the statistical analysis and its interpretation appropriate?

No

Are all the source data underlying the results available to ensure full reproducibility?

Yes

Is the study design appropriate and is the work technically sound?

Yes

Are the conclusions drawn adequately supported by the results?

Yes

Are sufficient details of methods and analysis provided to allow replication by others?

No

Reviewer Expertise:

Epidemiology, Environmental health, Biostatistical modeling

I confirm that I have read this submission and believe that I have an appropriate level of expertise to confirm that it is of an acceptable scientific standard, however I have significant reservations, as outlined above.

# Response

**Published:** 2024-06-22

**Authors:** Prajna Nayak

**Affiliations:** Manipal Academy of Higher Education, India

We would like to thank the reviewer for spending their valuable time and providing us with their valuable comments and suggestions. We have tried to address the comments and revised the manuscript accordingly. The changes in the manuscript have been highlighted in yellow.

**Reviewer Comment:**

**1. Population Size and Sampling Method:**

**Current Status: The revised methods section includes details on the small population size and the use of complete enumeration.**

**Recommendation 1: Explicitly state any limitations related to sample size, potential biases, and the generalizability of findings in a dedicated paragraph in the discussion section.**

**Recommendation 2: Finite Population Correction (FPC): If applicable, include the finite population correction factor in the descriptive statistical analysis section. This adjustment is crucial for small populations and should be transparently reported to strengthen the credibility of the findings.**

***Author Response:*** We thank the reviewer for the suggestion, we have now explicitly mentioned the same.

 Respected ma’am, as it was not possible to estimate the sample size, further incorporating the criteria of Finite Population Correction was not considered.

**Reviewer Comment:**

**2. Statistical Analysis Transparency:**

**T-Test and Chi-Square Test Assumptions:**

**T-Test: The manuscript now includes information on the assumptions for the t-test.**

**Chi-Square Test: The assumptions for the chi-square test, particularly the need for independence of observations and the requirement that expected frequencies be at least 5, have not been discussed.**

**Recommendation: Please discuss how the assumptions for both the t-test and chi-square test were verified. For the chi-square test, specifically confirm that all expected frequencies met the minimum requirement.**

***Author Response:***  We have now applied Fisher’s Exact Test as a replacement of Chi-square test, since the expected frequencies of few cells were less than 5. (Table 5).

 For table 4, where T-test was applied, we have mentioned the following in Page 6: ‘Since the sample was small, Shapiro Wilk test was done was performed, which did not show evidence of non-normality (W = 0.78, p-value = 0.26).’

**Reviewer Comment:**

**3. Confidence Intervals for Descriptive Statistics:**

**Current Status: The response indicates a misunderstanding about the applicability of confidence intervals for proportions. My suggestion was to estimate CIs for the descriptive statistics summarized in the first three tables, not specifically for hypothesis tests.**

**Recommendation: Confidence intervals should be calculated and presented for the descriptive statistics (proportions and means) summarized in Tables 1-3. This will enhance the interpretability and reliability of your estimates. Confidence intervals can and should be calculated for proportions and are a standard practice in statistical analysis to express the precision of an estimate. Providing confidence intervals for proportions, and not only means, would strengthen the presentation of your results by offering a range within which the true population proportion is likely to lie. This enhances the interpretability and reliability of your findings.**

**If there are specific reasons or constraints that will prevent you from including confidence intervals for proportions, it would be helpful to elaborate on these in the manuscript. Otherwise, I strongly recommend incorporating confidence intervals for the proportions presented in your results section to improve the completeness and robustness of your statistical analysis.**

***Author Response:***  Thank you, ma’am, for suggestion. We have now added Confidence Intervals for proportions. (Tables 1 & 2)

# Reviewer report: Reviewer response for version 3

**DOI:** 10.5256/f1000research.159529.r229119

**Published:** 2024-01-19

**Authors:** Nourdine Attiya

**Affiliations:** 1 Moulay Ismail University, Meknes, Morocco

Dear Authors’,

 I hope this message finds you well. Thank you for your continued efforts in revising the manuscript titled "Community social responsibility of continued and appropriate use of silver amalgam as dental restorative material in southern India: A cross-sectional study." I have thoroughly reviewed the revised version and would like to emphasize certain methodological concerns that, unfortunately, remain unaddressed:

**Methods:**

1. **Population Size and Sampling Method:**
  - The revised methods section still lacks essential information regarding the population size and the sampling method. This omission hampers the ability of readers to evaluate the generalizability of the study findings. Including these details is crucial for the overall transparency and robustness of your research.
2. **Statistical Analysis Transparency:**
  - I appreciate the clarity in your statistical analysis section. However, the revised manuscript does not mention whether all assumptions for the t-test and especially for the chi-square test were checked before application. Reporting on these checks and addressing any violations would significantly enhance the methodological transparency of your study.
  - The authors mentioned that perception and awareness for DA use as a risk factor were treated as continuous variables for analysis using Student's t-test. However, the manuscript lacks clarification on the specific transformation method employed to convert these variables into continuous form. Providing details on this transformation process would enhance the transparency and replicability of the study's statistical analysis
3. **Confidence Intervals:**
  - While proportions are presented in the results section, the absence of confidence intervals limits the precision of the estimates. Signaling this limitation in the discussion is not sufficient to accept it. Providing insights into the real reasons behind this absence is crucial and would strengthen the methodological rigor of your study.

On another hand, the revised version of the article has addressed some aspects of the initial concerns, but there are still areas that could be further refined:

1. **Neutrality and Balance:**
  - The authors have made slight revisions to the language, but there is room for improvement in achieving a more neutral tone.
2. **Inclusion of Opposing Views:**
  - The manuscript still lacks explicit inclusion of opposing viewpoints or critical studies on dental amalgam use. Enhancing the literature review with such perspectives would contribute to a more balanced overview.
3. **Clarity and Flow:**
  - While the manuscript maintains an acceptable reading flow, some sentences could be simplified for clarity. Revisiting sentence structures and avoiding unnecessary repetition would enhance communication.

While there has been some improvement, it seems that the initial concerns were not fully addressed in the revised submitted manuscript.

Is the work clearly and accurately presented and does it cite the current literature?

No

If applicable, is the statistical analysis and its interpretation appropriate?

No

Are all the source data underlying the results available to ensure full reproducibility?

Yes

Is the study design appropriate and is the work technically sound?

Yes

Are the conclusions drawn adequately supported by the results?

Yes

Are sufficient details of methods and analysis provided to allow replication by others?

No

Reviewer Expertise:

Epidemiology, Environmental health, Biostatistical modeling

I confirm that I have read this submission and believe that I have an appropriate level of expertise to state that I do not consider it to be of an acceptable scientific standard, for reasons outlined above.

# Response

**Published:** 2024-03-22

**Authors:** Prajna Nayak

**Affiliations:** Manipal Academy of Higher Education, India

Firstly, we would like to thank the reviewers for spending their valuable time and providing us with their valuable comments and suggestions. We truly appreciate your guidance in improving our work. We have tried to address the comments and revised the manuscript accordingly.

**Reviewer report and Author responses**

**1. Introduction:**

 Neutrality and Balance:

 The authors have made slight revisions to the language, but there is room for improvement in achieving a more neutral tone.    

**Response:** Respected ma’am, we have now revised achieve a more neutral tone.

 Inclusion of Opposing Views:

     The manuscript still lacks explicit inclusion of opposing viewpoints or critical studies on dental amalgam use. Enhancing the literature review with such perspectives would contribute to a more balanced overview.

**Response: **We thank the reviewer for the suggestion, we have now added 6 more recent studies, ma’am:

 No correlations were found between exposure to DA and neuropsychological and renal functions in children and adults in many randomized trials done all over the world. [Bellinger DC et al, 2006; Barregard L, Trachtenberg F and McKinlay S, 2008; Lauterbach M et al, 2008]. A systematic review and meta-analysis was conducted for three case control studies and one cohort study but did not provide evidence for or against an association between the presence of DA restorations and multiple sclerosis. [Aminzadeh KK, Etminan M, 2008].

 Studies did not find any association between urinary mercury concentrations among dentists and dental nurses with self-reported memory disturbance or mercury vapors in the dental office with cytogenetic damage to leukocytes. [Ritchie KA et al, 2004; Atesagaoglu A et al, 2004].

 Clarity and Flow:

     While the manuscript maintains an acceptable reading flow, some sentences could be simplified for clarity. Revisiting sentence structures and avoiding unnecessary repetition would enhance communication.

**Response: **We have now made the corrections, ma’am.

**3. Methods:**

 Population Size and Sampling Method:

     The revised methods section still lacks essential information regarding the population size and the sampling method. This omission hampers the ability of readers to evaluate the generalizability of the study findings. Including these details is crucial for the overall transparency and robustness of your research.

**Response: **Since it was a small population size, we included all practitioners, and hence, sampling was not done, ma’am.  Total population size (complete enumeration) is taken into the study. This is mentioned in the 2 ^nd^ and 5 ^th^ paragraphs of ‘Study design’ and 1 ^st^ paragraph of results section.

 (population size:134 dental practitioners, those following inclusion and exclusion criteria: 92, Responses: 52) Since it was a small population size, we have included all practitioners, and hence, no sampling was done.

  Statistical Analysis Transparency:

     I appreciate the clarity in your statistical analysis section. However, the revised manuscript does not mention whether all assumptions for the t-test and especially for the chi-square test were checked before application. Reporting on these checks and addressing any violations would significantly enhance the methodological transparency of your study.

     The authors mentioned that perception and awareness for DA use as a risk factor were treated as continuous variables for analysis using Student's t-test. However, the manuscript lacks clarification on the specific transformation method employed to convert these variables into continuous form. Providing details on this transformation process would enhance the transparency and replicability of the study's statistical analysis.      

**Response: **We regret the error in ‘statistical analysis section’ ma’am. We have now done the corrections.

 Perception and awareness for DA use as a risk factor were analyzed using chi-square test. (as seen in the table)

 Preferences for continuation of use of silver amalgam based upon the age of dentists had continuous variables (age), so analysis was done using Students t-test. (Table 4)

 All the other analysis were done using chi-square test, as they were proportions.

**4. Results:**

 Confidence Intervals:

     While proportions are presented in the results section, the absence of confidence interval limits the precision of the estimates. Signaling this limitation in the discussion is not sufficient to accept it. Providing insights into the real reasons behind this absence is crucial and would strengthen the methodological rigor of your study.

**Response: **Thank you, ma’am, since Confidence Intervals can be given for continuous variables, and not proportions, we have now added this in Table 4.

# Reviewer report: Reviewer response for version 2

**DOI:** 10.5256/f1000research.139800.r225243

**Published:** 2023-11-27

**Authors:** Nourdine Attiya

**Affiliations:** 1 Moulay Ismail University, Meknes, Morocco

I have had the opportunity to thoroughly review the research article titled:” **Community social responsibility of continued and appropriate use of silver amalgam as dental restorative material in southern India: A cross-sectional study**”, submitted for consideration to F1000Research and I would like to raise several points that, in my view, require careful consideration and clarification.

**1. Introduction:**

 The introduction provides a comprehensive historical background on dental amalgam, highlighting its significance and controversies. However, there seems to be a lack of neutrality in the language, particularly in statements like "We observed that DA is receiving undue attention and controversies." This may give the impression of bias and could be addressed for a more balanced tone.

**2. Literature Review:**

 While the literature review touches upon the controversies surrounding dental amalgam, it appears to be somewhat one-sided. It would be beneficial to include key studies or viewpoints that are critical of amalgam use to provide a more comprehensive overview of the current landscape. Moreover, I observed that the bibliography includes references that are somewhat dated. Given the dynamic nature of research in this field, it is essential to incorporate the most recent and pertinent literature to enhance the credibility and relevance of the study.

   **3.** **Methods:**

- The methods section is clear, but there is a lack of information regarding the population size and the sampling method. Including these details is crucial for readers to assess the generalizability of the study findings and understand how the sample was selected and if it is representative of the sought population.
- Additionally, the statistical analysis section does not mention whether assumptions for the t-test (normality, homoscedasticity) and chi-square test (expected frequencies in each cell are not too small, especially for variables with multiple categories) were checked before application. Reporting on these checks and how violations, if any, were addressed would enhance the transparency of the study.

** 4. Results:**

 The results section presents proportions without confidence intervals. The absence of confidence intervals limits the ability to gauge the precision of the estimates. In the discussion, it would be beneficial to acknowledge and explain this limitation.

**5. Discussion & conclusion:**

- While the discussion is comprehensive, it would be helpful to emphasize the strengths and positive aspects of dental amalgam, providing a more balanced view.
- The use of a specific commercial product name in the conclusion (Ivoclar Cention N) raises ethical considerations. Scientific research should prioritize objectivity, and care should be taken to avoid any perception of endorsing or promoting a specific company's product.
- The conclusion rightly emphasizes the need for a calculated decision in selecting the right restorative material. However, it would be beneficial to reiterate key recommendations (promoted by the Minamata convention and several organizations), such as the importance of education on mercury hygiene and waste management.

Finally, throughout the article, I think that clarity and conciseness could be enhanced by revisiting sentence structures and avoiding unnecessary repetition. Moreover, neutrality in presenting the dental amalgam controversial scientific debate is recommended.

Is the work clearly and accurately presented and does it cite the current literature?

No

If applicable, is the statistical analysis and its interpretation appropriate?

No

Are all the source data underlying the results available to ensure full reproducibility?

Yes

Is the study design appropriate and is the work technically sound?

Yes

Are the conclusions drawn adequately supported by the results?

Yes

Are sufficient details of methods and analysis provided to allow replication by others?

No

Reviewer Expertise:

Epidemiology, Environmental health, Biostatistical modeling

I confirm that I have read this submission and believe that I have an appropriate level of expertise to state that I do not consider it to be of an acceptable scientific standard, for reasons outlined above.

# Response

**Published:** 2023-12-06

**Authors:** Prajna Nayak

**Affiliations:** Manipal Academy of Higher Education, India

Firstly, we would like to thank the reviewer for spending their valuable time and providing us with their valuable comments and suggestions. We truly appreciate your guidance in improving our work. We have tried to address the comments and revised the manuscript accordingly.

**Reviewer report and Author responses**

 1. Introduction:

 The introduction provides a comprehensive historical background on dental amalgam, highlighting its significance and controversies. However, there seems to be a lack of neutrality in the language, particularly in statements like "We observed that DA is receiving undue attention and controversies." This may give the impression of bias and could be addressed for a more balanced tone.      

**Response:** Respected ma’am, we have now modified it.

 2. Literature Review:      

 While the literature review touches upon the controversies surrounding dental amalgam, it appears to be somewhat one-sided. It would be beneficial to include key studies or viewpoints that are critical of amalgam use to provide a more comprehensive overview of the current landscape. Moreover, I observed that the bibliography includes references that are somewhat dated. Given the dynamic nature of research in this field, it is essential to incorporate the most recent and pertinent literature to enhance the credibility and relevance of the study.     

**Response: **We have now added 3 more recent studies, ma’am. We have also attempted to give the dual stance of organizations and research works done and tried to explain both standpoints.

  3. Methods:     

     The methods section is clear, but there is a lack of information regarding the population size and the sampling method. Including these details is crucial for readers to assess the generalizability of the study findings and understand how the sample was selected and if it is representative of the sought population.

**Response: **Total population size is mentioned in the 2nd paragraph of ‘Study design’ (population size:134 dental practitioners).

 Since it was a small population size, we have included all practitioners, and hence, no sampling was done.

 Additionally, the statistical analysis section does not mention whether assumptions for the t-test (normality, homoscedasticity) and chi-square test (expected frequencies in each cell are not too small, especially for variables with multiple categories) were checked before application. Reporting on these checks and how violations, if any, were addressed would enhance the transparency of the study.              

**Response: **We have now added on the normality of the data.

 4. Results:         

 The results section presents proportions without confidence intervals. The absence of confidence intervals limits the ability to gauge the precision of the estimates. In the discussion, it would be beneficial to acknowledge and explain this limitation.           

**Response: **We have now mentioned ma’am

 5. Discussion & conclusion:         

     While the discussion is comprehensive, it would be helpful to emphasize the strengths and positive aspects of dental amalgam, providing a more balanced view.          

**Response: **We have briefly mentioned the strengths of dental amalgam in the 1st paragraph and limitations in the 3rd paragraph of discussion

    

 The use of a specific commercial product name in the conclusion (Ivoclar Cention N) raises ethical considerations. Scientific research should prioritize objectivity, and care should be taken to avoid any perception of endorsing or promoting a specific company's product.              

**Response: **Thank you for drawing attention towards this. We have now removed the brand names, ma’am.

    

 The conclusion rightly emphasizes the need for a calculated decision in selecting the right restorative material. However, it would be beneficial to reiterate key recommendations (promoted by the Minamata convention and several organizations), such as the importance of education on mercury hygiene and waste management.              

**Response: **We have now rectified it, ma’am.

 Finally, throughout the article, I think that clarity and conciseness could be enhanced by revisiting sentence structures and avoiding unnecessary repetition. Moreover, neutrality in presenting the dental amalgam controversial scientific debate is recommended.             

**Response: **We have now attempted and appraised, ma’am.

# Reviewer report: Reviewer response for version 2

**DOI:** 10.5256/f1000research.139800.r154244

**Published:** 2022-11-02

**Authors:** Manjunath P Puranik

**Affiliations:** 1 Department of Public Health Dentistry, Government Dental College and Research Institute, Bangalore, Karnataka, India

Revision is satisfactory.

Is the work clearly and accurately presented and does it cite the current literature?

Yes

If applicable, is the statistical analysis and its interpretation appropriate?

Yes

Are all the source data underlying the results available to ensure full reproducibility?

Yes

Is the study design appropriate and is the work technically sound?

Yes

Are the conclusions drawn adequately supported by the results?

Yes

Are sufficient details of methods and analysis provided to allow replication by others?

Yes

Reviewer Expertise:

Public Health Dentistry

I confirm that I have read this submission and believe that I have an appropriate level of expertise to confirm that it is of an acceptable scientific standard.

# Reviewer report: Reviewer response for version 1

**DOI:** 10.5256/f1000research.134713.r150726

**Published:** 2022-10-06

**Authors:** Manjunath P Puranik

**Affiliations:** 1 Department of Public Health Dentistry, Government Dental College and Research Institute, Bangalore, Karnataka, India

1. Abstract - " *In spite of satisfaction with DA for its minimal failure, longevity and affordability, the authors found that a majority of practitioners did not prefer its continued usage*." - There is a contradiction in their practice, perceptions and preferences. This has to be explained in the discussion.
2. Abstract - " *Dentists with higher age and longer clinical experience preferred continuation of DA.*" - Is it because of lack of awareness or any other reason? This has to be explained in the discussion.
3. Abstract - " *There is also an urgent need to educate dentists on mercury hygiene, mercury waste management and disposal.*" **- Will this help dentists with higher age and longer clinical experience who preferred continuation of DA? This has to be explained in the discussion.
4. Abstract - " *This highlights their concerns over mercury toxicity and soft tissue lesions and accentuates their community social responsibility." *- How does it accentuate their community social responsibility? This has to be explained in the discussion.
5. Introduction - is extensive and informative about the stance of dental amalgam usage by various organizations around the world. Since the study is among individual dentists, the review demands the addition of such information if there is any in the existing literature and justifies the need for the study.
6. Discussion - there is a need to discuss community social responsibility since it is mentioned in the title.

Is the work clearly and accurately presented and does it cite the current literature?

Yes

If applicable, is the statistical analysis and its interpretation appropriate?

Yes

Are all the source data underlying the results available to ensure full reproducibility?

Yes

Is the study design appropriate and is the work technically sound?

Yes

Are the conclusions drawn adequately supported by the results?

Yes

Are sufficient details of methods and analysis provided to allow replication by others?

Yes

Reviewer Expertise:

Public Health Dentistry

I confirm that I have read this submission and believe that I have an appropriate level of expertise to confirm that it is of an acceptable scientific standard, however I have significant reservations, as outlined above.
